# Supplementary material for: Reliable and Sensitive Nested PCR for the Detection of Chlamydia in Sputum
Source: Microorganisms. 2021 Apr 27;9(5):935. doi: 10.3390/microorganisms9050935 (PMC8145989; doi:10.3390/microorganisms9050935)
Supplement: Supplementary file 1 [file microorganisms-09-00935-s001.zip › microorganisms-1202603-Supplementary Materials.pdf]

Alignment statistics for match #1

Score	Expect	Identities	Gaps	Strand
441 bits(488)	2e-122	819/1190(69%)	28/1190(2%)	Plus/Minus

```
Query  1       ATGAAAAAACTCTTAAAGTCGGCGTTATTATCCGCCGCATTTGCTGGTTCTGTCGGCTCC  60
               |||||||||||||| || ||||  ||| |||  ||||| ||||   ||||||    ||||
Sbjct  785542  ATGAAAAAACTCTTGAAATCGGTATTAGTATTTGCCGC-TTTGA--GTTCTGCTTCCTCC  785486

Query  61      TTACAAGCCTTGCCTGTAGGGAACCCTTCTGATCCAAGCTTATTAATTGATGGTACAATA  120
               || |||||  ||||||| ||||| ||| |||| |||||| |  | || || || |   |
Sbjct  785485  TTGCAAGCTCTGCCTGTGGGGAATCCTGCTGAACCAAGCCTTATGATCGACGGAATTCTG  785426

Query  121     TGGGAAGGTGCTGCAGGAGATCCTTGCGATCCTTGCGCTACTTGGTGCGACGCTATTAGC  180
               |||||||||   |  |||||||||||||||||||| | |||||||| |||||||| |||
Sbjct  785425  TGGGAAGGTTTCGGCGGAGATCCTTGCGATCCTTGCACCACTTGGTGTGACGCTATCAGC  785366

Query  181     TTACGTGCTGGATTTTACGGAGACTATGTTTTCGACCGTATCTTAAAAGTAGATGCACCT  240
                | |||   || |  || || |||| ||||||||||||| | || |||  |||||     |
Sbjct  785365  ATGCGTATGGGTTACTATGGTGACTTTGTTTTCGACCGTGTTTTGAAAACAGATGTGAAT  785306

Query  241     AAAACATTTTCTATGGGAGCCAAGCCTACTGGATCCGC------TGCTGCAAACTATACT  294
               |||  |||    ||||| | |||||||||  |  |  |      |||| ||       ||
Sbjct  785305  AAAGAATTCCAAATGGGTGACAAGCCTACAAGTACTACAGGCAATGCTACAGCTCCAACC  785246

Query  295     ACTGCCGTAGATAGACCTAACCCGGCCTACAATAAGCATTTACACGATGCAGAGTGGTTC  354
               |||     ||  |||   || || || |||       ||| | || ||||| |||  |||
Sbjct  785245  ACTCTTACAGCAAGAGAGAATCCTGCTTACGGCCGACATATGCAGGATGCTGAGATGTTT  785186

Query  355     ACTAATGCAGGCTTCATTGCCTTAAACATTTGGGATCGCTTTGATGTTTTCTGTACTTTA  414
               || ||||| |  | ||| || || || ||||||||||||||||||| ||||||||  ||
Sbjct  785185  ACAAATGCCGCTTGCATGGCATTGAATATTTGGGATCGCTTTGATGTATTCTGTACACTA  785126

Query  415     GGAGCTTCTAATGGTTACATTAGAGGAAACTCTACAGCGTTCAATCTCGTTGGTTTATTC  474
               ||||| ||||  || ||| ||| |||||||||| |  | |||||| | ||||| || ||
Sbjct  785125  GGAGCCTCTAGCGGATACCTTAAAGGAAACTCTGCTTCTTTCAATTTAGTTGGATTGTTT  785066

Query  475     GGAGTTAAAGGTACT------ACTGTAAATGCAAATGAACTACCAAACGTTTCTTTAAGT  528
               |||| ||| |  | |      || || ||  | |||    |||||||  |    |||  |
Sbjct  785065  GGAGATAATGAAAATCAAAGCACGGTCAAAACGAATTCTGTACCAAATATGAGCTTAGAT  785006

Query  529     AACGGAGTTGTTGAACTTTACACAGACACCTCTTTCTCTTGGAGCGTAGGCGCTCGTGGA  588
                 |    ||||||||||||||||||| ||  | |||||||||||||| |||||||| | |
Sbjct  785005  CAATCTGTTGTTGAACTTTACACAGATACTGCCTTCTCTTGGAGCGTGGGCGCTCGAGCA  784946

Query  589     GCCTTATGGGAATGCGGTTGTGCAACTTTGGGAGCTGAATTCCAATATGCACAGTCCAAA  648
               || || ||||| ||||| ||||| ||||| || |||   ||||||| || || || |||
Sbjct  784945  GCTTTGTGGGAGTGCGGATGTGCGACTTTAGGGGCTTCTTTCCAATACGCTCAATCTAAA  784886

Query  649     CCTAAAGTTGAAGAACTTAATGTGATCTGTAACGTATCGCAATTCTCTGTAAACAAACCC  708
               |||||||| |||||| | || ||  ||||||||| | |  | ||  || | || || ||
Sbjct  784885  CCTAAAGTCGAAGAATTAAACGTTCTCTGTAACGCAGCTGAGTTTACTATCAATAAGCCT  784826

Query  709     AAGGGCTATAAAGGCGTTGCTTTCCC-CTTGCCAACAGACGCTGGCGTAGCAACAGCTAC  767
               || || |||  |||    |  ||||| |||||   || | || ||    |    |||| ||
Sbjct  784825  AAAGGATATGTAGGGCAAGAATTCCCTCTTGCACTCATA-GCAGGAACTGATGCAGCGAC  784767

Query  768     TGGAACAAAGTCTGCGACCATCAATTATCATGAATGGCAAGTAGGAGCCTCTCTATCTTA  827
                || || ||    |||   | ||  |||| ||||| ||||||| | |      |||| |||||
Sbjct  784766  GGGCACTAAAGATGCCTCTATTGATTACCATGAGTGGCAAGCAAGTTTAGCTCTCTCTTA  784707

Query  828     CAGACT-AAACTCTTTAGTGCCATACATTGGAGTACAATGGTCTCGAGCAACTTTTGATG  886
               |||| | ||   | || | | || |||||||||||  |||||||||||||| |||||||
Sbjct  784706  CAGATTGAATATGTTCACT-CCCTACATTGGAGTTAAATGGTCTCGAGCAAGTTTTGATG  784648

Query  887     CTGATAACATCCGCATTGCTCAGCCAAAACTACCTACAGC--TGTTTTAAACTTAACTGC  944
               | ||||  || || || || |||||||||   | |||||||   | ||| | |||  ||  |
Sbjct  784647  CCGATACGATTCGTATAGCCCAGCCAAAATCAGCTACAGCTATCTTTGATACT--ACCAC  784590

Query  945     ATGGAACCCTTCT-TTACTAGGAAATGCCACAGCATTGTCTACTACTGA---TTCGTTCT  1000
                   |||||  || || || |||  || |    |       |||    | ||    |  | ||
Sbjct  784589  GCTTAACCCAACTATTGCT-GGAGCTGGCGATGTGAAAGCTAGCGCAGAGGGTCAGCTCG  784531

Query  1001    CAGACTTCATGCAAATTGTTTCCTGTCAGATCAACAAGTTTAAATCTAGAAAAGCTTGTG  1060
                 |||    ||||||||| || ||||  ||   | |||||| | |||||||||||| |||| |
Sbjct  784530  GAGATACCATGCAAATCGTCTCCTTGCAATTGAACAAGATGAAATCTAGAAAATCTTGCG  784471
```

```
Query  1061    GAGTTACTGTAGGAGCTACTTTAGTTGATGCTGATAAATGGTCACTTACTGCAGAAGCTC  1120
               |  || | |||||| | ||| | || ||||| || ||||   || |||| |  ||  |||
Sbjct  784470  GTATTGCAGTAGGAACGACTATTGTAGATGCAGACAAATACGCAGTTACAGTTGAGACTC  784411

Query  1121    GTTTAATTAACGAGAGAGCTGCTCACGTATCTGGTCAGTTCAGATTCTAA  1170
               | || ||  | ||||||||||||||||||   ||   || ||| | ||||||
Sbjct  784410  GCTTGATCGATGAGAGAGCTGCTCACGTAAATGCACAATTCCGCTTCTAA  784361
```

Figure S1 Comparison of *Chlamydia pneumoniae* strain WBB major outer membrane protein (*ompA*) gene (Query; DQ358972.1) and *Chlamydia trachomatis* strain E-DK-20, major outer membrane protein (*ompA*) gene (Sbjct).

```
                                    PNEU S
C.abortus       TAGATGGAGAAAAGGGAATTCCACGTGTAGCGGTGAAATGCGTAGATATGTGGAAGAACA
C.psittaci      TAGATGGAGAAAAGGGAATTCCACGTGTAGCGGTGAAATGCGTAGATATGTGGAAGAACA
C.pneumoniae    TAGATGGGGAAAAGGGAATTCCACGTGTAGCGGTGAAATGCGTAGATATGTGGAAGAACA
C.trachomatis   TAGATGGAGAAAAGGGAATTTCACGTGTAGCGGTGAAATGCGTAGATATGTGGAAGAACA
E.coli          TCGTAGAGGGGGGTAGAATTCCAGGTGTAGCGGTGAAATGCGTAGAGATCTGGAGGAATA
Shigella        TCGTAGAGGGGGGTAGAATTCCAGGTGTAGCGGTGAAATGCGTAGAGATCTGGAGGAATA
Yersinia        TTGTAGAGGGGGGTAGAATTCCAGGTGTAGCGGTGAAATGCGTAGAGATCTGGAGGAATA
Salmonella      TTGTAGAGGGGGGTAGAATTCCAGGTGTAGCGGTGAAATGCGTAGAGATCTGGAGGAATA
C.jejuni        AGGGAGAGGCAGATGGAATTGGTGGTGTAGGGGTAAAATCCGTAGATATCACCAAGAATA
                  *  *  *      *****    ****** *** **** ****** **     * *** *
                      Short up
C.abortus       CCAGTGGCGAAGGCGCTTTTCTAATTTACACCTGACGCTAAGGCGCGAAAGCAAGGGGAG
C.psittaci      CCAGTGGCGAAGGCGCTTTTCTAATTTACACCTGACGCTAAGGCGCGAAAGCAAGGGGAG
C.pneumoniae    CCAGTGGCGAAGGCGCTTTTCTAATTTATACCTGACGCTAAGGCGCGAAAGCAAGGGGAG
C.trachomatis   CCAGTGGCGAAGGCGCTTTTCTAATTTATACCTGACGCTAAGGCGCGAAAGCAAGGGGAG
E.coli          CCGGTGGCGAAGGCGGCCCCCTGGACGAAGACTGACGCTCAGGTGCGAAAGCGTGGGGAG
Shigella        CCGGTGGCGAAGGCGGCCCCCTGGACGAAGACTGACGCTCAGGTGCGAAAGCGTGGGGAG
Yersinia        CCGGTGGCGAAGGCGGCCCCCTGGACAAAGACTGACGCTCAGGTGCGAAAGCGTGGGGAG
Salmonella      CCGGTGGCGAAGGCGGCCCCCTGGACAAAGACTGACGCTCAGGTGCGAAAGCGTGGGGAG
C.jejuni        CCCATTGCGAAGGCGATCTGCTGGAACTCAACTGACGCTAAGGCGCGAAAGCGTGGGGAG
                **  * *********    **          ******** *** ********  ******

                                                                  Chp up
C.abortus       CAAACAGGATTAGATACCCTGGTAGTCCTTGCCGTAAACGATGCATACTTGATGTGGATA
C.psittaci      CAAACAGGATTAGATACCCTGGTAGTCCTTGCCGTAAACGATGCATACTTGATATGGATA
C.pneumoniae    CAAACAGGATTAGATACCCTGGTAGTCCTTGCCGTAAACGATGCATACTTGATGTGGATG
C.trachomatis   CAAACAGGATTAGATACCCTGGTAGTCCTTGCCGTAAACGATGCATACTTGATGTGGATG
E.coli          CAAACAGGATTAGATACCCTGGTAGTCCACGCCGTAAACGATGTCGACTTGGAGGTTGTG
Shigella        CAAACAGGATTAGATACCCTGGTAGTCCACGCCGTAAACGATGTCGACTTGGAGGTTGTG
Yersinia        CAAACAGGATTAGATACCCTGGTAGTCCACGCCGTAAACGATGTCGACTTGGAGGTTGTG
Salmonella      CAAACAGGATTAGATACCCTGGTAGTCCACGCCGTAAACGATGTCGACTTGGAGGTTGTG
C.jejuni        CAAACAGGATTAGATACCCTGGTAGTCCACGCCCTAAACGATGTACACTAGTTGTTGG-G
                ****************************  *** *********   *** *   *
                                                                 Chtin

C.abortus       GTCTCAACCCTATCCGTGTCGTAGCTAACGCGTTAAGTATGCCGCCTGAGGAGTACACTC
C.psittaci      GTCTCAACCCTATCCGTGTCGTAGCTAACGCGTTAAGTATGCCGCCTGAGGAGTACACTC
C.pneumoniae    GTCTCAACCCCATCCGTGTCGGAGCTAACGTGTTAAGTATGCCGCCTGAGGAGTACACTC
C.trachomatis   GTCTCAACCCCATCCGTGTCGGAGCTAACGCGTTAAGTATGCCGCCTGAGGAGTACACTC
E.coli          CCCTTGAGGC-GTGGCTTCCGGAGCTAACGCGTTAAGTCGACCGCCTGGGGAGTACGGCC
Shigella        CCCTTGAGGC-GTGGCTTCCGGAGCTAACGCGTTAAGTCGACCGCCTGGGGAGTACGGCC
Yersinia        CCCTTGAGGC-GTGGCTTCCGGAGCTAACGCGTTAAGTCGACCGCCTGGGGAGTACGGCC
Salmonella      CCCTTGAGGC-GTGGCTTCCGGAGCTAACGCGTTAAGTCGACCGCCTGGGGAGTACGGCC
C.jejuni        ATGCTAGTCATCTCAGTAATGCAGCTAACGCATTAAGTGTACCGCCTGGGGAGTACGGTC
                            *   *   * ********  ******   ******* *******   *
                                    Short down

Ch.abortus      GCAAGGGTGAAACTCAAAAGAATTGACGGGGGCCCGCACAAGCAGTGGAGCATGTGGTTT
Ch.psittaci     GCAAGGGTGAAACTCAAAAGAATTGACGGGGGCCCGCACAAGCAGTGGAGCATGTGGTTT
C.pneumoniae    GCAAGGGTGAAACTCAAAAGAATTGACGGGGGCCCGCACAAGCAGTGGAGCATGTGGTTT
C.trachomatis   GCAAGGGTGAAACTCAAAAGAATTGACGGGGGCCCGCACAAGCAGTGGAGCATGTGGTTT
E.coli          GCAAGGTTAAAACTCAAATGAATTGACGGGGGCCCGCACAAGCGGTGGAGCATGTGGTTT
Shigella        GCAAGGTTAAAACTCAAATGAATTGACGGGGGCCCGCACAAGCGGTGGAGCATGTGGTTT
Yersinia        GCAAGGTTAAAACTCAAATGAATTGACGGGGGCCCGCACAAGCGGTGGAGCATGTGGTTT
Salmonella      GCAAGGTTAAAACTCAAATGAATTGACGGGGGCCCGCACAAGCGGTGGAGCATGTGGTTT
C.jejuni        GCAAGATTAAAACTCAAAGGAATAGACGGGGACCCGCACAAGCGGTGGAGCATGTGGTTT
                *****  * ********* **** ******* *********** ****************
```

```
C.abortus        AATTCGATGCAACGCGAAGAACCTTACCTGGGCTTGACATGTATTTGACCGCGGCAGAAA
C.psittaci       AATTCGATGCAACGCGAAGAACCTTACCTGGGCTTGACATGTATTTGACCGCGGCAGAAA
C.pneumoniae     AATTCGATGCAACGCGAAGGACCTTACCTGGACTTGACATGTATTTGACAACTGTAGAAA
C.trachomatis    AATTCGATGCAACGCGAAGGACCTTACCTGGGTTTGACATGTATATGACCGCGGCAGAAA
E.coli           AATTCGATGCAACGCGAAGAACCTTACCTGGTCTTGACATCCACGGAAGT-TTTCAGAGA
Shigella         AATTCGATGCAACGCGAAGAACCTTACCTGGTCTTGACATCCACAGAACC-TTGTAGAGA
Yersinia         AATTCGATGCAACGCGAAGAACCTTACCTGGTCTTGACATCCACAGAACT-TTCCAGAGA
Salmonella       AATTCGATGCAACGCGAAGAACCTTACCTGGTCTTGACATCCAGAGAACC-TGGCAGAGA
C.jejuni         AATTCGAAGATACGCGAAGAACCTTACCTGGGCTTGATATCCTAAGAACC-TTATAGAGA
                 ******* *  ******** ***********  **** **       *       *** *

C.abortus        TGTC--GTTTTC----CGCAAGGACAGATACACAGGTGCTGCATGGCTGTCGTCAGCTCG
C.psittaci       TGTC--GTTTTC----CGCAAGGACAGATACACAGGTGCTGCATGGCTGTCGTCAGCTCG
C.pneumoniae     TACA--GCTTTC----CGCAAGGACAGATACACAGGTGCTGCATGGCTGTCGTCAGCTCG
C.trachomatis    TGTC--GTTTTC----CACAAGGACATATACACAGGTGCTGCATGGCTGTCGTCAGCTCG
E.coli           TGAGAATGTGCC----TTCGGGAACCGTGAGACAGGTGCTGCATGGCTGTCGTCAGCTCG
Shigella         TACGAGGGTGCC----TTCGGGAACTGTGAGACAGGTGCTGCATGGCTGTCGTCAGCTCG
Yersinia         TGGATTGGTGCC----TTCGGGAACTGTGAGACAGGTGCTGCATGGCTGTCGTCAGCTCG
Salmonella       TGCCGGGGTGCC----TTCGGGAACTGTGAGACAGGTGCTGCATGGCTGTCGTCAGCTCG
C.jejuni         TATGAGGGTGCTAGCTTGCTAGAACTTAGAGACAGGTGCTGCACGGCTGTCGTCAGCTCG
                 *       *         *  * **    * ************ ****************

C.abortus        TGCCGTGAGGTGTTGGGTTAAGTCCCGCAACGAGCGCAACCCTTATCGTTAGTTGCCAAC
C.psittaci       TGCCGTGAGGTGTTGGGTTAAGTCCCGCAACGAGCGCAACCCTTATCGTTAGTTGCCAAC
C.pneumoniae     TGCCGTGAGGTGTTGGGTTAAGTCCCGCAACGAGCGCAACCCTTATCGTTAGTTGCCAGC
C.trachomatis    TGCCGTGAGGTGTTGGGTTAAGTCCCGCAACGAGCGCAACCCTTATCGTTAGTTGCCAGC
E.coli           TGTTGTGAAATGTTGGGTTAAGTCCCGCAACGAGCGCAACCCTTATCCTTTGTTGCCAGC
Shigella         TGTTGTGAAATGTTGGGTTAAGTCCCGCAACGAGCGCAACCCTTATCCTTTGTTGCCAGC
Yersinia         TGTTGTGAAATGTTGGGTTAAGTCCCGCAACGAGCGCAACCCTTATCCTTTGTTGCCAGC
Salmonella       TGTTGTGAAATGTTGGGTTAAGTCCCGCAACGAGCGCAACCCTTATCCTTTGTTGCCAGC
C.jejuni         TGTCGTGAGATGTTGGGTTAAGTCCCGCAACGAGCGCAACCCACGTATTTAGTTGCTAAC
                 **  ****  ********************************   *  ** ***** * *

C.abortus        ACTTAGGGTGGGAACTCTAACGAGACTGCCTGGGTTAACCAGGAGGAAGGCGAGGATGAC
C.psittaci       ACTTAGGGTGGGAACTCTAACGAGACTGCCTGGGTTAACCAGGAGGAAGGCGAGGATGAC
C.pneumoniae     ACTTAGGGTGGGAACTCTAACGAGACTGCCTGGGTTAACCAGGAGGAAGGCGAGGATGAC
C.trachomatis    ACTTAGGGTGGGAACTCTAACGAGACTGCCTGGGTTAACCAGGAGGAAGGCGAGGATGAC
E.coli           GGTCCGGCCGGGAACTCAAAGGAGACTGCCAGTGATAAACTGGAGGAAGGTGGGGATGAC
Shigella         GGTCCGGCCGGGAACTCAAAGGAGACTGCCAGTGATAAACTGGAGGAAGGTGGGGATGAC
Yersinia         GGTCCGGCCGGGAACTCAAAGGAGACTGCCAGTGATAAACTGGAGGAAGGTGGGGATGAC
Salmonella       GGTTCGGCCGGGAACTCAAAGGAGACTGCCGGTGATAAACCGGAGGAAGGTGGGGATGAC
C.jejuni         GGTTCGGCCGAGCACTCTAAATAGACTGCCTTCG-TAAGGAGGAGGAAGGTGTGGACGAC
                   *  **  * * **** **  ********   * ***   ********* * *** ***

C.abortus        GTCAAGTCAGCATGGCCCTTATGCCCAGGGCTACACACGTGCTACAATGGCCAGTACAGA
C.psittaci       GTCAAGTCAGCATGGCCCTTATGCCCAGGGCTACACACGTGCTACAATGGCCAGTACAGA
C.pneumoniae     GTCAAGTCAGCATGGCCCTTATGTCCAGGGCGACACACGTGCTACAATGGTTAGTACAGA
C.trachomatis    GTCAAGTCAGCATGGCCCTTATGCCCAGGGCGACACACGTGCTACAATGGCCAGTACAGA
E.coli           GTCAAGTCATCATGGCCCTTACGACCAGGGCTACACACGTGCTACAATGGCGCATACAAA
Shigella         GTCAAGTCATCATGGCCCTTACGACCAGGGCTACACACGTGCTACAATGGCGCATACAAA
Yersinia         GTCAAGTCATCATGGCCCTTACGACCAGGGCTACACACGTGCTACAATGGCGCATACAAA
Salmonella       GTCAAGTCATCATGGCCCTTACGACCAGGGCTACACACGTGCTACAATGGCGCATACAAA
C.jejuni         GTCAAGTCATCATGGCCCTTATGCCCAGGGCGACACACGTGCTACAATGGCATATACAAT
                 ********* *********** * ******* ******************    ****

C.abortus        AGGTAGCAATATCGCAAGATGGAGCAAATCCT-CAAAGCTGGCCCCAGTTCGGATTGTAG
C.psittaci       AGGTAGCAATATCGTGAGATGGAGCAAATCCT-CAAAGCTGGCCCCAGTTCGGATTGTAG
C.pneumoniae     AGGTAGCAAGATCGTGAGATGGAGCAAATCCT-AAAAGCTAGCCCCAGTTCGGATTGTAG
C.trachomatis    AGGTGGCAAGATCGCGAGATGGAGCAAATCCT-CAAAGCTGGCCCCAGTTCGGATTGTAG
E.coli           GAGAAGCGACCTCGCGAGAGCAAGCGGACCTCATAAAGTGCGTCGTAGTCCGGATTGGAG
Shigella         GAGAAGCGACCTCGCGAGAGCAAGCGGACCTCATAAAGTGCGTCGTAGTCCGGATTGGAG
Yersinia         GAGAAGCGAACTCGCGAGAGCAAGCGGACCTCATAAAGTGCGTCGTAGTCCGGATTGGAG
Salmonella       GAGAAGCGACCTCGCGAGAGCAAGCGGACCTCATAAAGTGCGTCGTAGTCCGGATTGGAG
C.jejuni         GAGACGCAATACCGCGAGGTGGAGCAAATCTA-TAAAATATGTCCCAGTTCGGATTGTTC
                   *  ** *   **  **    ***  * *    ***    * *  *** *******
```

Chp down

PNEU NOK

A.

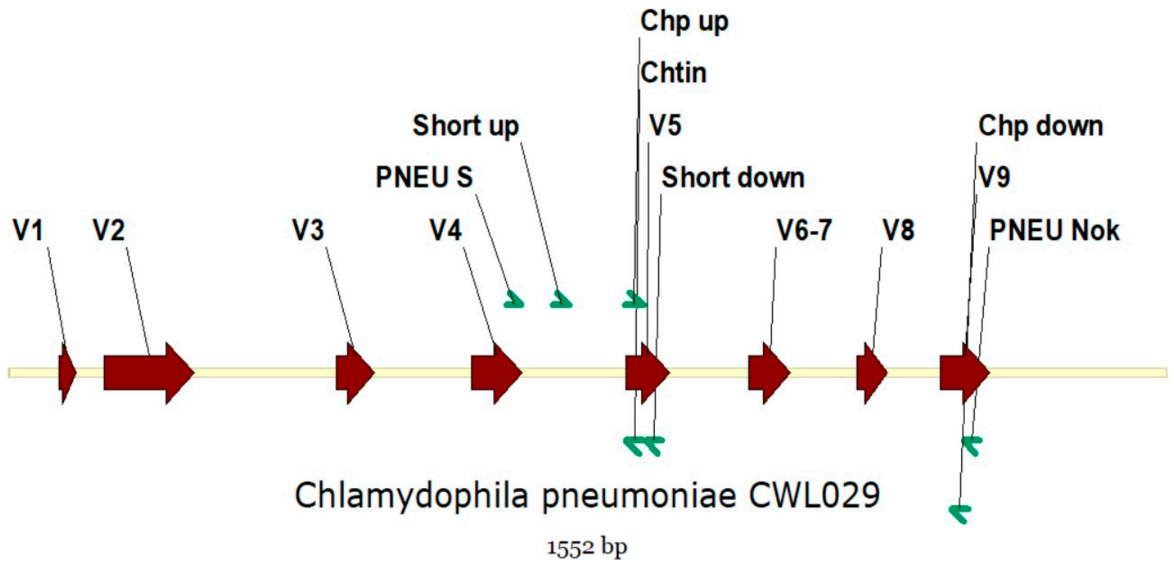

B.

Figure S2 Design of primers specific for four chlamydial species infecting humans. Reference sequences: *Chlamydia abortus* GN6 CP021996.1; *Chlamydia psittaci* 84/55 CP003790; *Chlamydia pneumoniae* TW-183 AE009440.1; *Chlamydia trachomatis* C/TW-3 CP006945.1; *E.coli* ECOUW87; *Shigella dysenteriae* CP061527.1; *Yersinia sp*. EU555393; *Salmonella enterica* CP045958.1; *Campylobacter jejuni* ATCC 35925 CP020045.1. **A**. Outer primers are in red letters and yellow shadowed; Inner primers are marked in blue; degenerate positions are grey shadowed. **B.** Location of the primers on the 16S rRNA gene. PNEU S pos. 676-686; PNEU Nok pos. 1280-1301; Chp up pos. 826-850; Chp down pos. 1264-1286; Short down pos. 854-874; Short up pos. 729-750; Chtinok pos. 826-850.

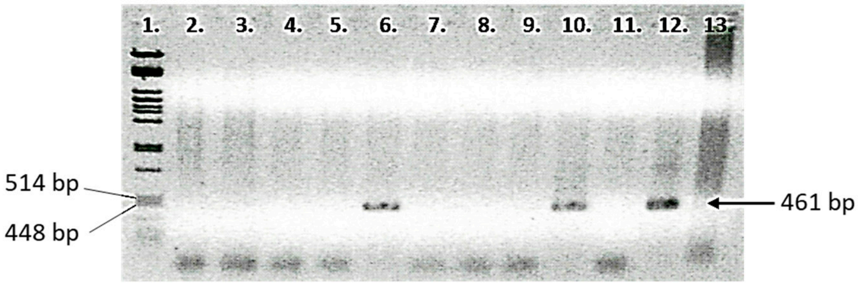

A.

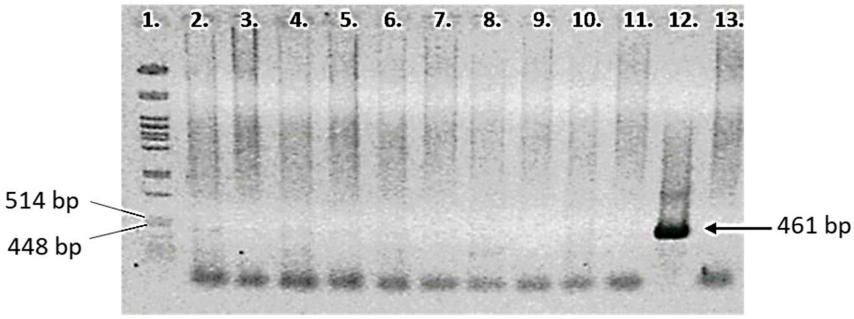

B.

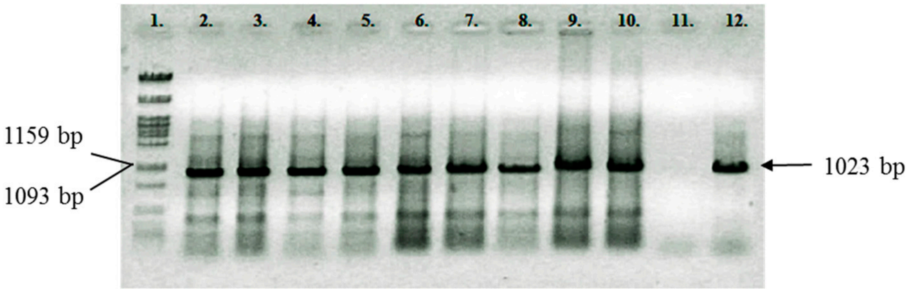

C.

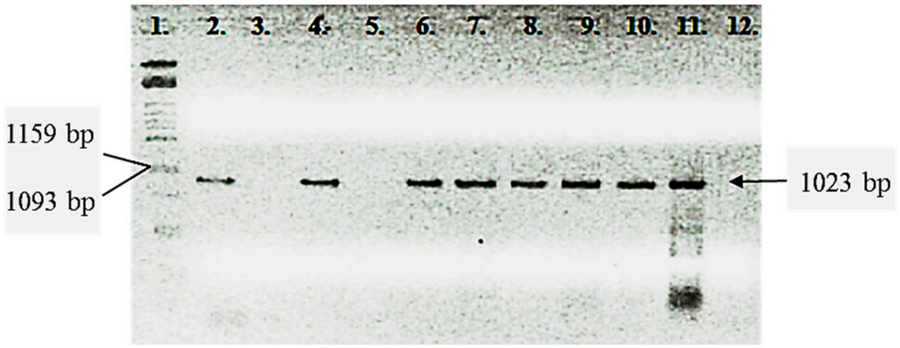

D.

Figure S3 Amplification and DNA degradation during 1 year of storage at -20°C. Lanes: 1. *λ*/*Pst*1 size marker, 2. 3SP, 3. NC, 4. 4SP, 5. NC, 6. 5SP, 7. NC. 8. 6SP, 9. NC, 10. 33SP, 11/12 positive and negative control (NC). **A. B.** Chp up/Chp down primers. Size of PCR product is 461 bp. **A**. Fresh DNA preps. **B**. After 1 year of storage at -20°C. **C. D.** MT for/MT rev primers. Size of PCR product is 1023 bp. **C.** Fresh DNA preps. **D.** After 1 year of storage at -20°C.

>DS
AGCAAGGGGAGCAAACAGGATTAGATACCCTGGTAGTCCTTGCCGTAAACGATCCATACTTG
ATGTGGATGGTCTCAAA

>MR
AGCAAGGGGAGCAAACAGGATTAGATACCCTGGTAGTCCTTGCCGTAAACGATCCATACTTG
ATGTGGATGRTCTCAAA

>2SP
AGCAAGGGGAGCAAACAGGATTRGATACCCTGGTAGTCCTTGCCGTAAACGATCCATACTTG
ATGTGGATGGTCTCAAA

>10SP
AGCAAGGGGAGCAAACAGGATTAGATACCCTGGTAGTCCTTGCCGTAAACGATCCATACTTG
ATGTGGATGGTCTCAAA

>18SP
AGCAAGGGGAGCAAACAGGATTAGATACCCTGGTAGTCCTTGCCGTAAACGATCCATACTTG
ATGTGGATGGTCTCAAA

>21SP
AGCAAGGGGAGCAAACAGGATTAGATACCCTGGTAGTCCTTGCCGTAAACGATCCATACTTG
ATGTGGATGGTCTCAAA

>28SP
AGCAAGGGGAGCAAACAGGATTAGATACCCTGGTAGTCCTTGCCGTAAACGATCCATACTTG
ATGTGGATGGTCTCAAA

>33SP
AGCAAGGGGAGCAAACAGGATTAGATACCCTGGTAGTCCTTGCCGTAAACGATCCATACTTG
ATGTGGATGGTcTCAAA

>45SP
AGCAAGGGGAGCAAACAGGATTAGATACCCTGGTAGTCCTTGCCGTAAACGATCCATACTTG
ATGTGGATGGTCTCAAA

>67SP
AGCAAGGGGAGCAAACAGGATTAGATACCCTGGTAGTCCTTGCCGTAAACGATCCATACTTG
ATGTGGATGGTCTCAAA

>3002
AGCAAGGGGAGCAAACAGGATTAGATACCCTGGTAGTCCTTGCCGTAAACGATCCATACTTG
ATGTGGATGGTCTCAAA

>3003
AGCAAGGGGAGCAAACAGGATTAGATACCCTGGTAGTCCTTGCCGTAAACGATCCATACTTG
ATGTGGATGGTCTCAAA

>3014
AGCAAGGGGAGCAAACAGGATTAGATACCCTGGTAGTCCTTGCCGTAAACGATCCATACTTG
ATGTGGATGGTCTCAAA

>3016
AGCAAGGGGAGCAAACAGGATTAGATACCCTGGTAGTCCTTGCCGTAAACGATCCATACTTG
ATGTGGATGGTCTCAAA

>3022
AGCAAGGGGAGCAAACAGGATTAGATACCCTGGTAGTCCTTGCCGTAAACGATCCATACTTG
ATGTGGATGGTCTCAAA

>3031
AGCAAGGGGAGCAAACAGGATTAGATACCCTGGTAGTCCTTGCCGTAAACGATCCATACTTG
ATGTGGATGGTCTCAAA

>5005
AGCAAGGGGAGCAAACAGGATTAGATACCCTGGTAGTCCTTGCCGTAAACGATCCATACTTG
ATGTGGATGGTCTCAAA

>5009
AGCAAGGGGAGCAAACAGGATTAGATACCCTGGTAGTCCTTGCCGTAAACGATCCATACTTG
ATGTGGATGGTCTCAAA

>7CHP
AGCAAGGGGAGCAAACAGGATTGGATACCCTGGTAGTCCTTGCCGTAAACGATCCATACTTG
ATGTGGATGGTCTCAAA

>13CHP
AGCAAGGGGAGCAAACAGGATTAGATACCCTGGTAGTCCTTGCCGTAAACGATCCATACTTG
ATGTGGATGGTCTCAAA

>14CHP
AGCAAGGGGAGCAAACAGGAATAGATACCCTGGTAGTCCTTGCCGTAAACGATCCATACTTG
ATGTGGATGGTCTCAAA

>16CHP
AGCAAGGGGAGCAAACAGGATTAGATACCCTGGTAGTCCTTGCCGTAAACGATCCATACTTG
ATGTGGATGGTCTCAAA

>18CHP
AGCAAGGGGAGCAAACAGGATTAGATACCCTGGTAGTCCTTGCCGTAAACGATCCATACTTG
ATGTGGATGGTCTCAAA

>28CHP
AGCAAGGGGAGCAAACAGGATTAGATACCCTGGTAGTCCTTGCCGTAAACGATCCATACTTG
ATGTGGATGGTCTCAAA

>41CHP
AGCAAGGGGAGCAAACAGGATTAGATACCCTGGTAGTCCTTGCCGTAAACGATCCATACTTG
ATGTGGATGGTCTCAAA

>45CHP
AGCAAGGGGAGCAAACAGGATTAGATACCCTGGTAGTCCTTGCCGTAAACGATCCATACTTG
ATGTGGATGGTCTCAAA

>48CHP
AGCAAGGGGAGCAAACAGGATTAGATACCCTGGTAGTCCTTGCCGTAAACGATCCATACTTG
ATGTGGATGGTCTCAAA

>49CHP
AGCAAGGGGAGCAAACAGGATTAGATACCCTGGTAGTCCTTGCCGTAAACGATCCATACTTG
ATGTGGATGGTCTCAAA

>58CHP
AGCAAGGGGAGCAAACAGGATTAGATACCCTGGTAGTCCTTGCCGTAAACGATCCATACTTG
ATGTGGATGGTCTCAAA

>60CHP
AGCAAGGGGAGCAAACAGGATTAGATACCCTGATAGTCCTTGCCGTAAACGATCCATACTTG
ATGTGGATGGTCTCAAA

>71CHP
AGCAAGGGGAGCAAACAGGATTAGATACCCTGGTAGTCCTTGCCGTAAACGATCCATACTTG
ATGTGGATGGTCTCAAA

>78CHP
AGCAAGGGGAGCAAACAGGATTAGATACCCTGGTAGTCCTTGCCGTAAACGATCCATACTTG
ATGTGGATGGTCTCAAA

>84CHP
AGCAAGGGGAGCAAACAGGATTAGATACCCTGGTAGTCCTTGCCGTAAACGATCCATACTTG
ATGTGGATGGTCTCAAA

>93CHP
AGCAAGGGGAGCAAACAGGATTAGATACCCTGGTAGTCCTTGCCGTAAACGATCCATACTTG
ATGTGGATGGTCTCAAA

>97CHP
AGCAAGGGGAGCAAACAGGATTAGATACCCTGGTAGTCCTTGCCGTAAACGATCCATACTTG
ATGTGGATGGTCTCAAA

>111CHP
AGCAAGGGGAGCAAACAGGATTAGATACCCTGGTAGTCCTTGCCGTAAACGATCCATACTTG
ATGTGGATGGTCTCAAA

>*C. trachomatis*
AGCAAGGGGAGCAAACAGGATTAGATACCCTGGTAGTCCTTGCCGTAAACGATGCATACTTG
ATGTGGATGGTCTCAA

>*C. pneumoniae*
AGCAAGGGGAGCAAACAGGATTAGATACCCTGGTAGTCCTTGCCGTAAACGATGCATACTTG
ATGTGGATGGTCTCAA

>*C. psittaci*

AGCAAGGGGAGCAAACAGGATTAGATACCCTGGTAGTCCTTGCCGTAAACGATGCATACTTG
ATGTGGATAGTCTCAA

>*C. abortus*

AGCAAGGGGAGCAAACAGGATTAGATACCCTGGTAGTCCTTGCCGTAAACGATGCATACTTG
ATGTGGATAGTCTCAA

A.

>DS
TATGCCGCCTGAGGAGTACACTCGCAAGGGTGAAACTCAAAAGAATTGACGGGGGCCCGCA
CAAGCAGTGGAGCATGTGGTTTAATTCGATGCAACGCGAAGGACCTTACCTGGACTTGACAT
GTATTTGACAACTGTAGAAATACAGCTTTCCGCAAGGACAGATACACAGGTGCTGCATGGCT
GTCGTCAGCTCGTGCCGTGAGGTGTTGGGTTAAGTCCCGCAACGAGCGCAACCCTTATCGTTA
GTTGCCAGCACTTAGGGTGGGAACTCTAACGAGACTGCCTGGGTTAACCAGGAGGAAGGCGA
GGATGACGTCAAGTCAGCATGGCCCTTATGTCCAGGGCGACACACGTGCTACAATGGTTAGT
ACAGAAGGTAGCAAGATC

>MR
TATGCCGCCTGAGGAGTACACTCGCAAGGGTGAAACTCAAAAGAATTGACGGGGGCCCGCA
CAAGCAGTGGAGCATGTGGTTTAATTCGATGCAACGCGAAGGACCTTACCTGGACTTGACAT
GTATTTGACAACTGTAGAAATACAGCTTTCCGCAAGGACAGATACACAGGTGCTGCATGGCT
GTCGTCAGCTCGTGCCGTGAGGTGTTGGGTTAAGTCCCGCAACGAGCGCAACCCTTATCGTTA
GTTGCCAGCACTTAGGGTGGGAACTCTAACGAGACTGCCTGGGTTAACCAGGAGGAAGGCGA
GGATGACGTCAAGTCAGCATGGCCCTTATGTCCAGGGCGACACACGTGCTACAATGGTTAGT
ACAGAAGGTAGCAAGATC

>PS
TATGCCGCCTGAGGAGTACACTCGCAAGGGTGAAACTCAAAAGAATTGACGGGGGCCCGCA
CAAGCAGTGGAGCATGTGGCTTAATTCGATGCAACGCGAAGGACCTTACCTGGACTTGACAT
GTATTTGACAACTGTAGAAATACAGCTTTCCGCAAGGACAGATACACAGGTGCTGCATGGCT
GTCGTCAGCTCGTGCCGTGAGGTGTTGGGTTAAGTCCCGCAACGAGCGCAACCCTTATCGTTA
GTTGCCAGCACTTAGGGTGGGAACTCTAACGAGACTGCCTGGGTTAACCAGGAGGAAGGCGA
GGATGACGTCAAGTCAGCATGGCCCTTATGTCCAGGGCGACACACGTGCTACAATGGTTAAT
ACAGAAGGTAGCAAGATC

>5SP
TATGCCGCCTGAGGAGTACACTCGCAAGGGTGAAACTCAAAAGAATTGACGGGGGCCCGCA
CAAGCAGTGGAGCATGTGGTTTAATTCGATGCAACGCGAAGGACCTTACCTGGACTTGACAT
GTATTTGACAACTGTAGAAATACAGCTTTCCGCAAGGACAGATACACAGGTGCTGCATGGCT
GTCGTCAGCTCGTGCCGTGAGGTGTTGGGTTAAGTCCCGCAACGAGCGCAACCCTTATCGTTA
GTTGCCAGCACTTAGGGTGGGAACTCTAACGAGACTGCCTGGGTTAACCAGGAGGAAGGCGA
GGATGACGTCAAGTCAGCATGGCCCTTATGTCCAGGGCGACACACGTGCTACAATGGTTAGT
ACAGAAGGTAGCAAGATC

>10SP
TATGCCGCCTGAGGAGTACACTCGCAAGGGTGAAACTCAAAAGAATTGACGGGGGCCCGCA
CAAGCAGTGGAGCATGTGGTTTAATTCGATGCAACGCGAAGGACCTTACCTGGACTTGACAT
GTATTTGACAACTGTAGAAATACAGCTTTCCGCAAGGACAGATACACAGGTGCTGCATGGCT
GTCGTCAGCTCGTGCCGTGAGGTGTTGGGTTAAGTCCCGCAACGAGCGCAACCCTTATCGTTA
GTTGCCAGCACTTAGGGTGGGAACTCTAACGAGACTGCCTGGGTTAACCAGGAGGAAGGCGA
GGATGACGTCAAGTCAGCATGGCCCTTATGTCCAGGGCGACACACGTGCTACAATGGTTAGT
ACAGAAGGTAGCAAGATC

>18SP
CATGCCGCCTGAGGAGTACACTCGCAAGGGTGAAACTCAAAAGAATTGACGGGGGCCCGCA
CAAGCAGTGGAGCATGTGGTTTAATTCGATGCAACGCGAAGGACCTTACCTGGACTTGACAT
GTATTTGACAACTGTAGAAATACAGCTTTCCGCAAGGACAGATACACAGGTGCTGCATGGCT
GTCGTCAGCTCGTGCCGTGAGGTGTTGGGTTAAGTCCCGCAACGAGCGCAACCCTTATCGTTA
GTTGCCAGCACTTAGGGTGGGAACTCTAACGAGACTGCCTGGGTTAACCAGGAGGAAGGCGA
GGATGACGTCAAGTCAGCATGGCCCTTATGTCCAGGGCGACACACGTGCTACAATGGTTAGT
ACAGAAGGTAGCAAGATC

>20SP
TATGCCGCCTGAGGAGTACACTCGCAAGGGTGAAACTCAAAAGAATTGACGGGGGCCCGCA
CAAGCAGTGGAGCATGTGGTTTAATTCGATGCAACGCGAAGGACCTTACCTGGACTTGACAT
GTATTTGACAACTGYAGAAATACAGCTTTCCGCAAGGACAGATACACAGGTGCTGCATGGCT
GTCGTCAGCTCGTGCCGTGAGGTGTTGGGTTAAGTCCCGCAACGAGCGCAACCCTTATCGTTA
GTTGCCAGCACTTAGGGTGGGAACTCTAACGAGACTGCCTGGGTTAACCAGGAGGAAGGCGA
GGATGACGTCAAGTCAGCATGGCCCTTATGTCCAGGGCGACACACGTGCTACAATGGTTAGA
TCAGAAGGTAGCAAGATC

>21SP
TATGCCGCCTGAGGAGTACACTCGCAAGGGTGAAACTCAAAAGAATTGACGGGGGCCCGCA
CAAGCAGTGGAGCATGTGGTTTAATTCGATGCAACGCGAAGGACCTTACCTGGACTTGACAT
GTATTTGACAACTGTAGAAATACAGCTTTCCGCAAGGACAGATACACAGGTGCTGCATGGCT
GTCGTCAGCTCGYGCCGTGAGGTGTTGGGTTAAGTCCCGCAACGAGCGCAACCCTTATCGTTA
GTTGCCAGCACTTAGGGTGGGAACTCTAACGAGACTGCCTGGGTTAACCAGGGGAAGGCGAG
GATGACGTCAAGTCAGCATGGCCCTTATGTCCAGGGCGACACACGTGCTACAATGGTTAGTAC
AGAAGGTAGCAAGATC

>33SP
TATGCCGCCTGAGGAGTACACTCGCAAGGGTGAAACTCAAAAGAATTGACGGGGGCCCGCA
CAAGCAGTGGAGCATGTGGTTTAATTCGATGCAACGCGAAGGACCTTACCTGGACTTGACAT
GTATTTGACAACTGTAGAAATACAGCTTTCCGCAAGGACAGATACACAGGTGCTGCATGGCT
GTCGTCAGCTCGTGCCGTGAGGTGTTGGGTTAAGTCCCGCAACGAGCGCAACCCTTATCGTTA
GTTGCCARCACTTAGGGTGGGAACTCTAACGAGACTGCCTGGGTTAACCAGGAGGAAGGCGA
GGATGACGTCAAGTCAGCATGGCCCTTATGTCCAGGGCGACACACGTGCTACAATGGTTAGT
ACAGAAGGTAGCAAGATC

>45SP
TATGCCGCCTGAGGAGTACACTCGCAAGGGTGAAACTCAAAAGAATTGACGGGGGCCCGCA
CAAGCAGTGGAGCATGTGGTTTAATTCGATGCAACGCGAAGGACCTTACCTGGACTTGACAT
GTATTTGACAACTGTAGAAATACAGCTTTCCGCAAGGACAGATACACAGGTGCTGCATGGCT
GTCGTCAGCTCGTGCCGTGAGGTGTTGGGTTAAGTCCCGCAACGAGCGCAACCCTTATCGTTA
GTTGCCAGCACTTAGGGTGGGAACTCTAACGAGACTGCCTGGGTTAACCAGGAGGAAGGCGA
GGATGACGTCAAGTCAGCATGGCCCTTATGTCCAGGGCGACACACGTGCTACAATGGTTAGT
ACAGAAGGTAGCAAGATC

>3014
TACTCAAGAATTGGACGGGCGTGTTCCGCACAAGCAGTGGAGCATGTGGTTTAATTCGATGCA
ACGCGAAGAACCTTACCCAGGCTCGAAATGCAAAGGCAATCAACAGAGATGTTGAGTTCCGC
AAGGACTTTTGTATAGGTGCTGCATGGCTGTCGTCAGCTCGTGCCGTGAGGTGTTGGGTTAAGT
CCCGCAACGAGCGCAACCCTTATCGTTAGTTGCCAACATTTAGGTGGGAACTCTAACGAGAC
TGCCTGGGTTAACCAGGAGGAAGGTGAGGATGACGTCAAGTCCGCATGGCCTTTATGTCTGG

GGCTACACACGTGCTACAATGGTCGGTACAGAAGGCAGCAAGATCGTGAGATGGAGCAAATCA

>3016
TATGCCGCCTGAGGAGTACACTCGCAAGGGTGAAACTCAAAAGAATTGACGGGGGCCCGCACAAGCAGTGGAGCATGTGGTTTAATTCGATGCAACGCGAAGGACCTTACCTGGACTTGACATGTATTTGACAACTGTAGAAATACAGCTTTCCGCAAGGACAGATACACAGGTGCTGCATGGCTGTCGTCAGCTCGTGCCGTGAGGTGTTGGGTTAAGTCCCGCAACGAGCGCAACCCTTATCGTTAGTTGCCAGCACTTAGGGTGGGAACTCTAACGAGACTGCCTGGGTTAACCAGGAGGAAGGCGAGGATGACGTCAAGTCAGCATGGCCCTTATGTCCAGGGCGACACACGTGCTACAATGGTTAGTACAGAAGGTAGCAAGATC

>57CHP
TGGGGAGTACGCTCGCAAGGGTGAAACTCAAAAGAATTGACGGGGGCCCGCACAAGCAGTGGAGCATGTGGTTTAATTCGATGCAACGCGAAGAACCTTACCCAGGCTCGAAATGCAAAGGCAATCAACAGAGATGTTGAGTTCCGAAAGGACTTTTGTATAGGTGCTGCATGGCTGTCGTCAGCTCGTGCCGTGAGGTGTTGGGTTAAGTCCCGCAACGAGCGCAACCCTTATCGTTAGTTGCCAACACGCAAGGTGGGGACTCTAACGAGACTGCCTAGGTTAACCTAGGAGGAAGGTGAGGATGACGTCAAGTCCGCATGGCCTTTATGTCTGGGGCTACACA

B.

Figure S4 A. Sequences of 79 nt SNPCR products, **B**. sequences of 346 nt of SNPCR product amplified from sputa.

CLUSTAL X (1.81) multiple sequence alignment

```
14.CHP             AGCAAGGGGAGCAAACAGGAATAGATACCCTGGTAGTCCTTGCCGTAAACGATCCATACT
60.CHP             AGCAAGGGGAGCAAACAGGATTAGATACCCTGATAGTCCTTGCCGTAAACGATCCATACT
2.SP               AGCAAGGGGAGCAAACAGGATTRGATACCCTGGTAGTCCTTGCCGTAAACGATCCATACT
7.CHP              AGCAAGGGGAGCAAACAGGATTGGATACCCTGGTAGTCCTTGCCGTAAACGATCCATACT
MR                 AGCAAGGGGAGCAAACAGGATTAGATACCCTGGTAGTCCTTGCCGTAAACGATCCATACT
111.CHP            AGCAAGGGGAGCAAACAGGATTAGATACCCTGGTAGTCCTTGCCGTAAACGATCCATACT
97.CHP             AGCAAGGGGAGCAAACAGGATTAGATACCCTGGTAGTCCTTGCCGTAAACGATCCATACT
93.CHP             AGCAAGGGGAGCAAACAGGATTAGATACCCTGGTAGTCCTTGCCGTAAACGATCCATACT
84.CHP             AGCAAGGGGAGCAAACAGGATTAGATACCCTGGTAGTCCTTGCCGTAAACGATCCATACT
78.CHP             AGCAAGGGGAGCAAACAGGATTAGATACCCTGGTAGTCCTTGCCGTAAACGATCCATACT
71.CHP             AGCAAGGGGAGCAAACAGGATTAGATACCCTGGTAGTCCTTGCCGTAAACGATCCATACT
58.CHP             AGCAAGGGGAGCAAACAGGATTAGATACCCTGGTAGTCCTTGCCGTAAACGATCCATACT
49.CHP             AGCAAGGGGAGCAAACAGGATTAGATACCCTGGTAGTCCTTGCCGTAAACGATCCATACT
48.CHP             AGCAAGGGGAGCAAACAGGATTAGATACCCTGGTAGTCCTTGCCGTAAACGATCCATACT
45.CHP             AGCAAGGGGAGCAAACAGGATTAGATACCCTGGTAGTCCTTGCCGTAAACGATCCATACT
41.CHP             AGCAAGGGGAGCAAACAGGATTAGATACCCTGGTAGTCCTTGCCGTAAACGATCCATACT
28.CHP             AGCAAGGGGAGCAAACAGGATTAGATACCCTGGTAGTCCTTGCCGTAAACGATCCATACT
18.CHP             AGCAAGGGGAGCAAACAGGATTAGATACCCTGGTAGTCCTTGCCGTAAACGATCCATACT
16.CHP             AGCAAGGGGAGCAAACAGGATTAGATACCCTGGTAGTCCTTGCCGTAAACGATCCATACT
13.CHP             AGCAAGGGGAGCAAACAGGATTAGATACCCTGGTAGTCCTTGCCGTAAACGATCCATACT
5009.SP            AGCAAGGGGAGCAAACAGGATTAGATACCCTGGTAGTCCTTGCCGTAAACGATCCATACT
5005.SP            AGCAAGGGGAGCAAACAGGATTAGATACCCTGGTAGTCCTTGCCGTAAACGATCCATACT
3031.SP            AGCAAGGGGAGCAAACAGGATTAGATACCCTGGTAGTCCTTGCCGTAAACGATCCATACT
3022.SP            AGCAAGGGGAGCAAACAGGATTAGATACCCTGGTAGTCCTTGCCGTAAACGATCCATACT
3016.SP            AGCAAGGGGAGCAAACAGGATTAGATACCCTGGTAGTCCTTGCCGTAAACGATCCATACT
3014.SP            AGCAAGGGGAGCAAACAGGATTAGATACCCTGGTAGTCCTTGCCGTAAACGATCCATACT
3003.SP            AGCAAGGGGAGCAAACAGGATTAGATACCCTGGTAGTCCTTGCCGTAAACGATCCATACT
3002.SP            AGCAAGGGGAGCAAACAGGATTAGATACCCTGGTAGTCCTTGCCGTAAACGATCCATACT
67.SP              AGCAAGGGGAGCAAACAGGATTAGATACCCTGGTAGTCCTTGCCGTAAACGATCCATACT
45.SP              AGCAAGGGGAGCAAACAGGATTAGATACCCTGGTAGTCCTTGCCGTAAACGATCCATACT
33.SP              AGCAAGGGGAGCAAACAGGATTAGATACCCTGGTAGTCCTTGCCGTAAACGATCCATACT
28.SP              AGCAAGGGGAGCAAACAGGATTAGATACCCTGGTAGTCCTTGCCGTAAACGATCCATACT
21.SP              AGCAAGGGGAGCAAACAGGATTAGATACCCTGGTAGTCCTTGCCGTAAACGATCCATACT
18.SP              AGCAAGGGGAGCAAACAGGATTAGATACCCTGGTAGTCCTTGCCGTAAACGATCCATACT
10.SP              AGCAAGGGGAGCAAACAGGATTAGATACCCTGGTAGTCCTTGCCGTAAACGATCCATACT
DS                 AGCAAGGGGAGCAAACAGGATTAGATACCCTGGTAGTCCTTGCCGTAAACGATCCATACT
C.trachomatis      AGCAAGGGGAGCAAACAGGATTAGATACCCTGGTAGTCCTTGCCGTAAACGATGCATACT
C.pneumoniae       AGCAAGGGGAGCAAACAGGATTAGATACCCTGGTAGTCCTTGCCGTAAACGATGCATACT
C.psittaci         AGCAAGGGGAGCAAACAGGATTAGATACCCTGGTAGTCCTTGCCGTAAACGATGCATACT
C.abortus          AGCAAGGGGAGCAAACAGGATTAGATACCCTGGTAGTCCTTGCCGTAAACGATGCATACT
                   ******************** * ********* ******************** ******
```

```
14.CHP            TGATGTGGATGGTCTCAAA
60.CHP            TGATGTGGATGGTCTCAAA
2.SP              TGATGTGGATGGTCTCAAA
7.CHP             TGATGTGGATGGTCTCAAA
MR                TGATGTGGATGRTCTCAAA
111.CHP           TGATGTGGATGGTCTCAAA
97.CHP            TGATGTGGATGGTCTCAAA
93.CHP            TGATGTGGATGGTCTCAAA
84.CHP            TGATGTGGATGGTCTCAAA
78.CHP            TGATGTGGATGGTCTCAAA
71.CHP            TGATGTGGATGGTCTCAAA
58.CHP            TGATGTGGATGGTCTCAAA
49.CHP            TGATGTGGATGGTCTCAAA
48.CHP            TGATGTGGATGGTCTCAAA
45.CHP            TGATGTGGATGGTCTCAAA
41.CHP            TGATGTGGATGGTCTCAAA
28.CHP            TGATGTGGATGGTCTCAAA
18.CHP            TGATGTGGATGGTCTCAAA
16.CHP            TGATGTGGATGGTCTCAAA
13.CHP            TGATGTGGATGGTCTCAAA
5009.SP           TGATGTGGATGGTCTCAAA
5005.SP           TGATGTGGATGGTCTCAAA
3031.SP           TGATGTGGATGGTCTCAAA
3022.SP           TGATGTGGATGGTCTCAAA
3016.SP           TGATGTGGATGGTCTCAAA
3014.SP           TGATGTGGATGGTCTCAAA
3003.SP           TGATGTGGATGGTCTCAAA
3002.SP           TGATGTGGATGGTCTCAAA
67.SP             TGATGTGGATGGTCTCAAA
45.SP             TGATGTGGATGGTCTCAAA
33.SP             TGATGTGGATGGTCTCAAA
28.SP             TGATGTGGATGGTCTCAAA
21.SP             TGATGTGGATGGTCTCAAA
18.SP             TGATGTGGATGGTCTCAAA
10.SP             TGATGTGGATGGTCTCAAA
DS                TGATGTGGATGGTCTCAAA
C.trachomatis     TGATGTGGATGGTCTCAAA
C.pneumoniae      TGATGTGGATGGTCTCAAA
C.psittaci        TGATGTGGATAGTCTCAAA
C.abortus         TGATGTGGATAGTCTCAAA
                  **********  *******
```

Figure S5 Alignment of SNPCR sequences. Polymorphic sites are grey shadowed.

TABLE S1 Sputum sample data.

Number	Sample	Age	Gender	NPCR 461 bp	NPCR 121 bp	mtDNA 1023 bp	mtDNA 250 bp
1	DS*	27	F	+	+	+	nd
2	MŠ	24	F	-	-	+	nd
3	BŠ	24	F	-	-	+	nd
4	MS	27	M	-	-	+	nd
5	PS*	60	M	+	+	+	nd
6	MR	24	M	+	+	+	nd
7	2SP	24	F	-	+	+	nd
8	3SP	25	F	-	-	+	nd
9	4SP	27	F	-	-	+	nd
10	5SP	28	M	+	+	+	nd
11	6SP	55	F	-	-	+	nd
12	7SP	58	F	-	-	+	nd
13	10SP	27	F	+	+	-	+
14	11SP	60	F	-	-	+	nd
15	17SP	28	F	-	-	+	nd
16	18SP	30	M	+	+	+	nd

17	20SP	26	F	+	+	+	nd
18	21SP	27	F	+	+	+	nd
19	27SP	22	F	-	-	+	nd
20	28SP	55	M	-	+	+	nd
21	29SP	35	M	-	-	+	nd
22	30SP	53	M	-	-	+	nd
23	33SP	23	F	+	+	+	nd
24	34SP	27	M	-	-	+	nd
25	37SP	49	F	-	-	+	nd
26	38SP	49	M	-	-	+	nd
27	39SP	22	F	-	-	+	nd
28	41SP	65	M	-	-	+	nd
29	44SP	65	M	-	-	+	nd
30	45SP*	31	M	+	+	+	nd
31	48SP	53	F	-	-	+	nd
32	49SP	72	M	-	-	+	nd
33	52SP	29	F	-	-	+	nd
34	54SP	22	F	-	-	+	nd
35	58SP	26	M	-	-	+	nd
36	60SP	22	M	-	-	+	nd
37	61SP	26	F	-	-	+	nd
38	64SP	23	F	-	-	+	nd
39	66SP	20	F	-	-	+	nd
40	67SP	20	F	-	+	+	nd
41	68SP	26	F	-	-	+	nd
42	69SP	40	F	-	-	+	nd
43	70SP	31	M	-	-	+	nd
44	1002	41	M	-	-	+	nd
45	3001	25	M	-	-	+	nd
46	3002	25	M	-	+	+	nd
47	3003	25	M	-	+	+	nd
48	3004	24	M	-	-	+	nd
49	3005	25	M	-	-	+	nd
50	3006	22	M	-	-	+	nd
51	3007	28	F	-	-	+	nd
52	3008	30	M	-	-	+	nd
53	3009	20	M	-	-	+	nd
54	3010	22	M	-	-	+	nd
55	3011	26	M	-	-	+	nd
56	3012	21	M	-	-	+	nd
57	3013	28	M	-	-	+	nd
58	3014	22	M	+	+	+	nd
59	3015	27	M	-	-	+	nd
60	3016	27	M	+	+	+	nd
61	3017	30	M	-	-	+	nd
62	3018	21	M	-	-	+	nd
63	3019	30	M	-	-	+	nd
64	3020	30	M	-	-	+	nd
65	3021	19	M	-	-	+	nd

66	3022	21	M	-	+	+	nd
67	3023	24	M	-	-	+	nd
68	3024	24	M	-	-	+	nd
69	3025	24	M	-	-	-	+
70	3026	22	M	-	-	+	nd
71	3027	21	M	-	-	-	+
72	3028	26	M	-	-	+	nd
73	3029	24	M	-	-	+	nd
74	3030	23	F	-	-	+	nd
75	3031	26	M	-	+	+	nd
76	3032	21	M	-	-	+	nd
77	3033	30	M	-	-	+	nd
78	5002	63	F	-	-	+	nd
79	5003	57	F	-	-	+	nd
80	5004	51	M	-	-	+	nd
81	5005	77	M	-	+	+	nd
82	5006	74	F	-	-	+	nd
83	5007	66	M	-	-	+	nd
84	5008	51	F	-	-	+	nd
85	5009	66	F	-	+	+	nd
86	2CHP	24	M	-	-	-	+
87	3CHP	60	F	-	-	+	nd
88	4CHP	41	F	-	-	-	+
89	5CHP	59	M	-	-	-	+
90	7CHP	77	M	-	+	+	nd
91	9CHP	63	F	-	-	+	nd
92	11CHP	29	M	-	-	+	nd
93	13CHP	44	F	-	+	-	+
94	14CHP	47	M	-	+	+	nd
95	15CHP	47	M	-	-	-	+
96	16CHP	26	F	-	+	+	nd
97	18CHP	28	M	-	+	+	nd
98	19CHP	25	M	-	-	+	nd
99	20CHP	53	F	-	-	+	nd
100	21CHP	42	F	-	-	-	+
101	23CHP	27	M	-	-	+	nd
102	24CHP	33	F	-	-	+	nd
103	27CHP	48	M	-	-	+	nd
104	29CHP	44	F	-	-	+	nd
105	30CHP	55	F	-	-	+	nd
106	31CHP	66	M	-	-	+	nd
107	33CHP	69	M	-	-	+	nd
108	36CHP	62	F	-	-	+	nd
109	37CHP	67	M	-	-	-	+
110	38CHP	43	M	-	-	+	nd
111	39CHP	39	F	-	-	+	nd
112	41CHP	61	F	-	+	+	nd
113	45CHP	16	F	-	+	+	nd
114	48CHP	37	M	-	+	+	nd

115	49CHP	37	F	-	+	+	nd
116	52CHP	30	M	-	-	-	+
117	53CHP	55	F	-	-	+	nd
118	57CHP	43	F	+	+	+	nd
119	58CHP	67	F	-	+	+	nd
120	60CHP	22	M	-	+	-	+
121	62CHP	60	M	-	-	+	nd
122	63CHP	27	F	-	-	+	nd
123	64CHP	24	F	-	-	+	nd
124	71CHP	28	M	-	+	+	nd
125	73CHP	33	M	-	-	-	+
126	74CHP	56	F	-	-	+	nd
127	76CHP	29	M	-	-	+	nd
128	77CHP	33	M	-	-	-	+
129	78CHP	60	F	-	+	+	nd
130	80CHP	34	M	-	-	-	+
131	81CHP	38	F	-	-	+	nd
132	84CHP	60	F	-	+	+	nd
133	85CHP	61	M	-	-	+	nd
134	88CHP	49	M	-	-	+	nd
135	91CHP	37	F	-	-	+	nd
136	92CHP	43	M	-	-	-	+
137	93CHP	70	F	-	+	+	nd
138	96CHP	45	F	-	-	+	nd
139	97CHP	27	F	-	+	-	+
140	98CHP	44	F	-	-	-	+
141	101CHP	25	F	-	-	+	nd
142	102CHP	26	M	-	-	-	+
143	105CHP	34	F	-	-	+	nd
144	107CHP	23	F	-	-	-	+
145	108CHP	25	F	-	-	+	nd
146	109CHP	52	F	-	-	+	nd
147	110CHP	58	M	-	-	+	nd
148	111CHP	-	M	-	+	+	nd
149	1080*	24	M	+	-	+	nd
150	1082*	47	F	-	+	+	nd
151	1089*	65	F	-	+	+	nd
152	1096*	41	F	-	+	+	nd
153	1779*	33	F	-	+	+	nd
154	1788*	38	F	-	+	+	nd
155	1803*	52	M	-	+	+	nd
156	1828*	49	F	-	+	+	nd
157	1838*	42	M	-	+	+	nd

* positive or treshold IgG or IgA; nd – non determined
